# Acute Kidney Injury in Children: A Focus for the General Pediatrician

**DOI:** 10.3390/children11081004

**Published:** 2024-08-16

**Authors:** Giulio Rivetti, Pietro Gizzone, Delfina Petrone, Anna Di Sessa, Emanuele Miraglia del Giudice, Stefano Guarino, Pierluigi Marzuillo

**Affiliations:** Department of Woman, Child and of General and Specialized Surgery, Università degli Studi della Campania “Luigi Vanvitelli”, Via Luigi de Crecchio 2, 80138 Naples, Italy; giulio.rivetti@studenti.unicampania.it (G.R.); pietro.gizzone@studenti.unicampania.it (P.G.); delfina.petrone@studenti.unicampania.it (D.P.); anna.disessa@unicampania.it (A.D.S.); emanuele.miraglia@unicampania.it (E.M.d.G.); stefano.guarino@policliniconapoli.it (S.G.)

**Keywords:** acute kidney injury, chronic kidney disease, children, biomarkers

## Abstract

Acute kidney injury (AKI) presents significant challenges in pediatric care, often remaining underrecognized. This paper provides an overview of pediatric AKI, highlighting its epidemiology, pathophysiology, diagnosis, predisposing conditions, and treatment. AKI in children stems from diverse causes, including renal tubular damage, vasoconstriction, and inflammation. Diagnosis relies on traditional markers such as serum creatinine and urine output, alongside emerging biomarkers such as Cystatin C, NGAL, KIM-1, IL-18, TIMP-2 and IGFBP7, urinary calprotectin, URBP4, L-FABP, and clusterin. Various pediatric conditions predispose to AKI, including type 1 diabetes, pneumonia, bronchiolitis, appendicitis, gastroenteritis, COVID-19, multisystem inflammatory syndrome, sickle cell disease, and malignancies. Treatment entails supportive care with fluid management and, in severe cases, renal replacement therapy. Timely recognition and management are essential to mitigating adverse outcomes. Enhanced awareness and integration of novel biomarkers could improve pediatric AKI care, warranting further research for better diagnosis and management.

## 1. Introduction

Acute kidney injury (AKI) represents a pathological state marked by a rapid decline in kidney function, leading to a decrease in estimated glomerular filtration rate (eGFR), an accumulation of urea and other nitrogenous waste products, and an imbalance in extracellular fluid and electrolytes [1].

Kidney Disease/Improving Global Outcomes (KDIGO) defines AKI as either an increase in serum creatinine levels or a reduction in urine output [2]. KDIGO guidelines also differentiate between “hospital-acquired” AKI (HA-AKI), which occurs after 48 h of hospital admission, and “community-acquired” AKI (CA-AKI), which is present at the time of admission or develops within the first 48 h [2,3].

The kidneys are often the target of many systemic pathological processes, which, if not treated promptly, can lead to AKI. In the current literature, it has been reported that 1–25% of admissions to the intensive care unit (ICU) and 1–7% of all hospital admissions are indeed complicated by AKI [4]. AKI can lead to health consequences in children, both in the short and long term [5]. In fact, in the short term, AKI has been described as an independent risk factor for prolonged ICU stays, a longer duration of mechanical ventilation, and increased mortality among critically ill patients [6,7,8,9,10,11]. On the other hand, AKI is also well known to lead to long-term complications, increasing the risk of developing hypertension [12] and, even in the case of a mild episode, doubling the risk of chetonic kidney disease (CKD) [13].

AKI is often under-recognized in childhood [14] despite its non-negligible impact on kidney health, especially from a long-term perspective [13]. This review aims to elucidate the main characteristics of AKI in children, providing essential information for general pediatricians and practitioners to help them identify and manage AKI effectively.

## 2. Epidemiology of AKI in Childhood

In recent decades, the global epidemiology of pediatric AKI has been better understood due to standardized definitions, reliable biomarkers, national registries, and multinational studies [15,16]. Before the early 2000s, there were limited data on the incidence of AKI in children. A study conducted at a tertiary care center found that 227 children underwent dialysis over 8 years, resulting in an incidence rate of 0.8 per 100,000 in the general population [17]. Andreoli et al. observed an increase in AKI incidence among children in 2009, although specific rates were not provided [18]. The AWARE study, which included 4683 children, was the first multinational study on pediatric AKI, reporting an incidence rate of 26.9%, with 11.6% progressing to severe AKI [16].

The median age for AKI diagnosis is 10.8 years, with a notable proportion occurring in adolescents aged 15 to 18 years. Males experience a higher prevalence of AKI at 56.4%, contrasting with a balanced distribution of sexes in non-AKI hospitalizations (50.5% versus 49.5%) [19].

Research by Plumb et al. [20] indicated higher AKI episodes in younger children, with increased ICU admissions in both the youngest (1–5 years) and oldest (16–18 years) age groups. However, comprehensive global data on AKI in children, especially from middle- and low-income countries, remain scarce [21,22,23,24,25]. The impact of AKI is particularly severe in developing nations, where limited resources hinder the management of advanced kidney failure and the need for renal replacement therapy.

**Figure 1:**
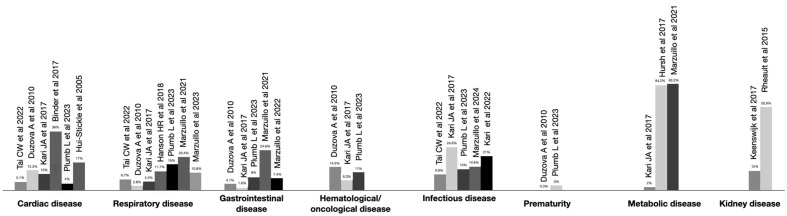
Causes and prevalence of AKI in children hospitalized for several diseases (from references [6,20,26,27,28,29,30,31,32,33,34,35,36,37,38,39,40].

The main causes of AKI in children are shown in Figure 1 [6,20,26,27,28,29,30,31,32,33,34,35,36,37,38,39,40].

## 3. Pathophysiology of AKI in Children 

AKI is defined by increased serum creatinine and nitrogenous waste products caused by poor organ perfusion, kidney cell damage, or acute obstruction of urine flow [18,41]. Children with a congenital reduced nephron population, due to conditions such as intrauterine growth restriction, antenatal glucocorticoid administration, or prematurity, are at higher risk for AKI [42]. 

AKI can be classified into pre-renal, renal, and post-renal categories based on the underlying cause. Pre-renal AKI is often due to dehydration and hypovolemia, as the kidneys play a critical role in maintaining body fluid and effective circulating volume [43,44]. Conditions such as vomiting, hemorrhage, diarrhea, poor water intake, burns, cardiorenal syndrome type 1, cardiac and non-cardiac surgery, and sepsis can lead to a functional pre-renal AKI [43,44]. 

Intrinsic renal AKI occurs due to diseases affecting the tubules, glomeruli, interstitium, and intrarenal blood vessels, with causes including acute tubular necrosis, tumor lysis syndrome, interstitial nephritis, glomerulonephritis, hemolytic uremic syndrome, and renal hypoplasia [45]. 

Finally, the post-kidney mechanism of AKI consists of an acute blockage of urine flow by obliterating one or both ureters at any level. In post-kidney AKI, the drop in glomerular filtration rate (GFR) is caused by a reduction of the intra-glomerular pressure determined by the increase in the tubular proximal pressure, which in turn is caused by the obstruction itself [45]. 

Reassuming that AKI can stem from various origins, despite sharing a common pathophysiological process [1].

The common pathway of pre-renal AKI involves compensatory mechanisms aimed at maintaining renal perfusion and ensuring adequate blood flow to the kidneys. One of the primary compensatory mechanisms is vasoconstriction, which occurs in response to decreased renal perfusion pressure [46]. When there is a decrease in renal perfusion pressure, the body activates the renin-angiotensin-aldosterone system (RAAS) [47]. Angiotensin II is a potent vasoconstrictor that acts on the efferent arterioles of the kidneys, causing them to constrict [47]. This vasoconstriction increases the resistance to blood flow in the efferent arterioles, leading to a decrease in renal blood flow [46]. However, it also results in an increase in glomerular capillary pressure, thereby helping to maintain GFR despite reduced renal perfusion pressure [46]. Additionally, angiotensin II stimulates the release of aldosterone, which promotes sodium and water reabsorption [48]. This retention of sodium and water helps to expand blood volume and maintain blood pressure, further supporting renal perfusion. However, while these compensatory mechanisms initially help to maintain renal function in response to decreased perfusion pressure, prolonged activation of these pathways can contribute to renal injury [46]. Persistent vasoconstriction and activation of the RAAS can lead to renal ischemia, tubular injury, and, ultimately, progression to intrinsic renal AKI if perfusion is not restored promptly [1].

Some authors divide AKI pathophysiology into four stages: initiation, extension, maintenance, and recovery [45]. During initiation, different mechanisms of AKI cause renal tubular damage, which leads to a decrease in the GFR, resulting in the apoptosis and necrosis of renal tubular cells [45]. Additionally, the renal insult triggers an inflammatory response, leading to the extension phase, which is clinically distinguished by a slower decline in GFR [45]. The stage known as maintenance starts instead when the GFR is steady and tubular and capillary cell repair begins [45]. Finally, the recovery phase is characterized by the restoration of tubular and capillary structures and by the recovery of kidney function, characterized by the rise in GFR [45]. 

## 4. How to Diagnose AKI

According to KDIGO, AKI can be defined by an increase in serum creatinine or a reduction in urine output, classifying AKI into three stages based on the degree of these changes (see KDIGO guidelines) [2].

The guidelines emphasize the importance of baseline serum creatinine (bSCr) values [2], which may be obtained from historical data or estimated using growth charts for children. However, the baseline serum creatinine is unknown in the majority of hospitalized children. The frequently utilized method for determining estimated baseline serum creatinine (ebSCr) involves retroactively calculating it using eGFR equations, assuming that basal eGFR are the median age-based eGFR normative values for children ≤ 2 years of age [49], and eGFR = 120 mL/min/1.73 m^2^ for children > 2 years [50]. When creatinine is measured by Jaffe method, the formula to calculate the ebSCr is expressed as ebSCr (mg/dL) = (k × height [cm])/120 mL/min/1.73 m^2^, where k equals 0.55 for female children and adolescents, and k equals 0.7 for male adolescents. The validity of the eGFR equation has been previously confirmed in the literature within cohorts of patients with an average age of 3.9 [51] years and 9.9 years [52].

It is advisable to utilize the updated Schwartz equation [53] for retrospectively calculating creatinine based on height when the creatinine measurement is conducted using the IDMS-traceable method [ebSCr (mg/dL) = (0.413 x height [cm])/(baseline eGFR)]. Nevertheless, if the Jaffe technique is utilized to measure creatinine, it is recommended to apply the original Schwartz equation [52,54].

## 5. Time-Honored Biomarkers of AKI

According to KDIGO, AKI is defined by increased serum creatinine or reduced urine output, but these markers have limitations [2]. Serum creatinine is a delayed indicator of kidney dysfunction, taking 24–48 h to accumulate in the blood to detectable levels [55,56]. It is also an indirect and unreliable GFR indicator influenced by age, sex, renal tubular secretion, fluid balance, muscle mass, and medications [55,56].

Furthermore, urinary output is unreliable in polyuric patients, such as those with type 1 diabetes mellitus (T1DM), where hyperglycemia and glycosylated hemoglobin can falsely elevate measured creatinine [52].

The fractional excretion of sodium (FENa) can be used as a parameter to distinguish pre-renal injury from hypoxic/ischemic AKI [18]. In fact, the tubules react to reduced renal perfusion during pre-renal injury by saving water and sodium so that the FENa < 1% and urinary sodium is less than 10–20 mEq/L [18]. When renal tubules have a sustained injury, they are unable to properly preserve water and salt, resulting in urinary sodium levels above 30–40 mEq/L and FENa > 2.0% [18].

## 6. Novel Biomarkers of AKI

Considering the limitations of currently used markers, which are increased after significant kidney damage, alternative functional and damage biomarkers of AKI, such as Cystatin C (Cys-C), Neutrophil Gelatinase-Associated Lipocalin (NGAL), Human-Kidney Injury Molecule-1 (KIM-1), Interleukin-18 (IL-18), Tissue inhibitor metalloproteinase 2 (TIMP-2) and Insulin-like Growth Factor Binding Protein 7 (IGFBP7), urinary calprotectin, Retinol binding protein (URPB4), Liver-type fatty acid binding protein (L-FABP), and clusterin, were evaluated. 

These biomarkers have been proven to provide valuable insights into the onset and progression of AKI in children, enabling quicker and more accurate diagnosis and personalized treatment. Their utilization facilitates the early identification of at-risk patients and aids in tracking disease progression, allowing healthcare providers to implement timely therapeutic interventions [57]. By guiding treatment decisions and enabling personalized care, biomarkers could enhance overall clinical outcomes and the quality of care in pediatric AKI management, contributing to improved patient prognosis and treatment efficacy.

Nevertheless, novel biomarkers are limitedly used due to the need for clinical validation, concerns regarding specificity and sensitivity, challenges related to cost and availability, the importance of standardization, and regulatory approval requirements [57].

However, ongoing research in this field offers promise for enhancing the early diagnosis, monitoring, and management of AKI in children.

Cys-C is a cysteine protease inhibitor, filtered by the glomerulus, fully reabsorbed, and not secreted at all [58]. Urinary excretion of Cys-C indicates tubular damage [59]. Altered levels of Cys-c can be a sign of acute and chronic kidney disease, and unlike creatinine, it does not depend on height, weight, age, sex, nutritional status, or inflammatory processes [59]. Nonetheless, Cys-C seems to function more as an indicator of GFR than a specific biomarker for AKI. Indeed, the precise role of Cys-C in diagnosing AKI remains unclear, primarily because of the fluctuating GFR levels observed during acute illness [60].

Other elements such as thyroid irregularities, obesity, corticosteroid usage, and inflammation may disrupt its serum levels [61].

NGAL is a component of innate immunity to bacterial infections and is expressed by immune cells, hepatocytes, and renal tubular cells in various disease states [62]. NGAL derives from proximal tubular cells and is detected in the urine at the early stage of AKI as an indicator of proximal tubular damage [61]. Urinary and/or circulating levels of NGAL are increased in the urine or serum within 6 h of the kidney injury [63]. NGAL levels appear to be more sensitive and specific in predicting AKI and with better accuracy in children than in adults [64]. The baseline plasma NGAL levels are elevated in individuals with malignancies and systemic bacterial infections, which can lead to confusion. Similarly, elevated levels of urinary NGAL may indicate urinary tract infections. Urinary NGAL serves as a diagnostic tool for the early detection of urinary tract infections, particularly when acute kidney injury is not present [61].

KIM-1 is an epithelial cell adhesion molecule expressed at a low level in normal kidneys but increased in the post-ischemic kidney, particularly in pre-renal kidney injury and after its reperfusion [61]. KIM-1 is synthesized soon after kidney injury; in fact, it was proven to be an effective biomarker of acute tubular damage [61]. The expression of renal KIM-1 was notably higher in human kidney tissue among patients diagnosed with focal glomerulosclerosis, immunoglobulin A nephropathy, membranoproliferative glomerulonephritis, membranous glomerulonephritis, acute rejection, chronic allograft nephropathy, systemic lupus erythematosus, diabetic nephropathy, hypertension, and Wegener’s granulomatosis compared to healthy kidney tissue. Urinary KIM-1 has demonstrated high sensitivity and specificity as a marker for proximal tubular kidney injury [61].

IL-18 is a proinflammatory cytokine expressed in the intercalated cells of the distal convolute tubule and the collecting tubule in the healthy human kidney [65]. Urinary levels of IL-18 have high sensitivity and specificity in identifying acute tubular necrosis, and an early increase in IL-18 levels correlates with the severity of AKI [61]. Viewing IL-18 as a proinflammatory substance crucial in the context of sepsis, its levels can be impacted by various factors, including inflammatory and autoimmune conditions. Elevations in serum IL-18 are observed in inflammatory arthritis, inflammatory bowel disease, systemic lupus erythematosus, psoriasis, hepatitis, and multiple sclerosis [61].

TIMP2 and IGFBP7: during the early stages of cellular stress, TIMP2 and IGFBP7 play a role in the G1 cell-cycle arrest phase. This G1 cell-cycle arrest phase is also experienced by renal tubular cells following stress brought on by a range of insults. Cell-cycle arrest signaling is a protective response by multiple cells and is detectable in urine following AKI; therefore, urinary TIMP-2*IGFBP-7 is considered an early marker for AKI [66]. Nevertheless, these biomarkers may be elevated in patients with diabetes [67].

Urinary calprotectin: calprotectin is a low-molecular-weight protein composed of two subunits (S100A8 and S100A9) that bind calcium and zinc, which are primarily released by neutrophils and monocytes in response to inflammatory processes [68]. Several studies have shown a significant increase in urinary calprotectin levels in patients with AKI compared to those without AKI [58,69,70]. Moreover, urinary calprotectin levels are significantly higher in patients with intrinsic AKI compared to those with prerenal AKI [58,69]. 

URPB4: URPB4 is among the most sensitive markers for detecting renal tubular damage [70]. Research by Varghese et al. has shown that by analyzing URBP4 and urinary albumin with a sophisticated algorithm, accurate diagnoses of AKI can be achieved [71]. Additionally, it has been observed that URBP4 levels normalize more rapidly than serum creatinine concentrations during recovery [72].

L-FABP: L-FABP is a protein involved in fatty acid transport at the cellular level, primarily found in the kidneys and liver [68]. Urinary L-FABP shows promise as a biomarker for detecting and evaluating AKI [73]. Ferguson et al. conducted a cross-sectional study indicating that urinary L-FABP serves as a robust biomarker for AKI and holds potential for predicting survival without the need for dialysis [74].

Clusterin: clusterin is a protein found in trace amounts in normal kidneys, primarily localized in the intima of renal arteries and renal tubules [75]. During AKI, clusterin expression increases, predominantly exhibiting anti-apoptotic effects and correlating with lipid utilization, cell aggregation, and adhesion [75]. Moreover, clusterin has been identified as one of the earliest markers of proximal tubular injury, with levels increasing within an hour of injury onset, well before serum creatinine elevation [76]. This underscores its potential as a promising marker for detecting both tubular and glomerular injuries.

These biomarkers could be useful in various clinical situations. For example, in patients in the ICU, new biomarkers can help identify AKI early in critically ill patients, allowing for timely interventions to prevent progressive renal damage. Additionally, after major surgical interventions, especially those with a high risk of AKI (such as cardiac surgery), biomarkers can be used to monitor renal function and quickly detect any signs of damage. New biomarkers also have diagnostic utility in patients with AKI and polyuria, such as children with diabetic ketoacidosis (DKA). Moreover, in patients with DKA, creatinine measurements might be overestimated by the presence of ketones [52].

The main advantages and disadvantages of the markers of AKI are highlighted in Table 1.

## 7. AKI Classification on the Basis of the Novel Biomarkers

Considering its innovative biomarkers, AKI can be categorized into subclinical and hemodynamic AKI. Subclinical AKI pertains to individuals with elevated tubular biomarkers but with preserved kidney function. On the other hand, hemodynamic AKI characterizes a syndrome prevalent in patients experiencing kidney functional loss despite the absence of evident kidney damage based on biomarkers, such as in cases of hypovolemia [77]. 

**Figure 2:**
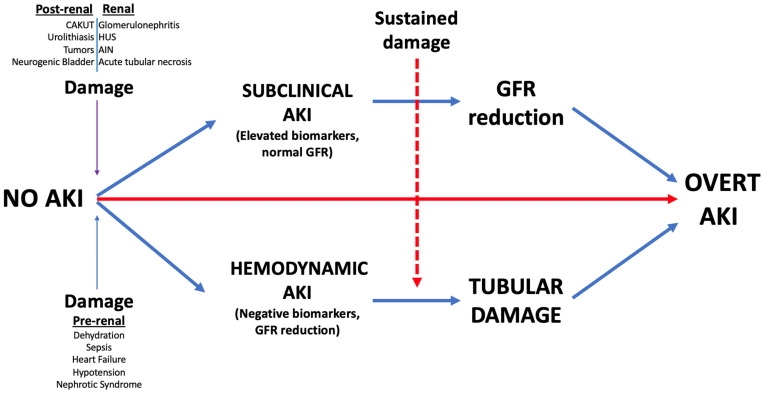
Classification and pathophysiology of AKI on the basis of the novel biomarkers.

Despite the possibility of renal injury initially manifesting as either subclinical or hemodynamic AKI, continued exposure to the insult can lead to the development of overt AKI, marked by diminished GFR and the presence of renal damage indicators (Figure 2).

In fact, as highlighted in Section 3, AKI often arises from diverse causes but typically shares a fundamental pathophysiological process. The decline in GFR, marking the initiation phase, serves as the common factor in this pathway. It can result from ischemic damage, as seen in pre-renal AKI, the obstruction of urinary flow leading to elevated intratubular pressure in post-renal AKI, or a variety of injuries affecting tubules, glomeruli, interstitium, and intra-renal blood vessels in intrinsic AKI.

Combining structural injury biomarkers (e.g., NGAL, TIMP2*IGFBP7) with functional biomarkers (e.g., SCr, Cystatin C) can improve the accuracy of diagnosing AKI. 

Increased SCr levels signify a decline in GFR, either in response to hemodynamic shifts or as a result of intrinsic kidney injury. Moreover, when injury biomarkers rise without a concurrent decline in renal function, it could suggest subclinical AKI, potentially indicating mild or compensated renal damage. If the detrimental insult persists, both scenarios have the potential to progress to overt AKI.

## 8. AKI in the Most Common Pediatric Conditions

AKI could be present in many common pediatric conditions (Figure 1), such as:

Metabolic diseases: children with type 1 diabetes mellitus (T1DM) may have kidney involvement both in the acute and chronic settings of the disease [78]. In fact, during T1DM onset, the osmotic polyuria caused by hyperglycemia leads to dehydration, hypovolemia, and kidney hypoperfusion, which in turn cause tubular damage. So then, during T1DM onset, AKI may occur in 43.8% of patients [32], and, separately evaluating patients with DKA, the prevalence increases up to 65% [32]. In fact, acidosis can accelerate the deterioration of tubular damage [78]. Nevertheless, the prevalence of AKI varies among patients with T1DM, as evidenced by different sources. For example, Hursh et al. demonstrated that among 165 retrospectively enrolled pediatric patients with DKA, 64% presented with AKI [33], while Khalifah et al. observed that, out of 213 patients admitted with DKA, 80.75% developed AKI.

Moreover, Pittmann et al. found that AKI occurred in 47% of cases, with a higher prevalence among those with DKA (83% in DKA patients versus 35% in non-DKA patients) [79]. Finally, Weissbach reported that among 82 children admitted to the Pediatric Intensive Care Unit (PICU) with DKA, 30% experienced AKI [80], and Baalaaji et al. similarly reported a prevalence of 35% of AKI in DKA patients admitted to the PICU [81].

Respiratory diseases: AKI may be a complication of respiratory conditions. For example, in hospitalized children affected by community-acquired pneumonia (CAP), AKI has been reported to occur with a prevalence of 20.4% [34]. Significant and independent predictors of AKI in children with CAP may be the duration of symptoms before hospitalization, the severity of pneumonia, and serum C-reactive protein levels [34]. The main pathophysiological mechanism linking AKI and CAP appears to be the systemic inflammatory response [34]. Another respiratory disease that may complicate AKI is acute bronchiolitis; indeed, about 11% of children hospitalized for viral bronchiolitis develop AKI [35]. Preterm birth and a birth weight less than the 10th percentile (conditions associated with a reduced nephronic mass) have been shown to be significantly associated with AKI development [35]. Moreover, dehydration has a role in the pathophysiology of AKI in children affected by viral bronchiolitis, since a close association between hematocrit >2SDS and AKI development has been discovered [35]. Furthermore, many cases of AKI have been described during COVID-19 infection; approximately one-fifth of the hospitalized patients develop an AKI during COVID-19 infection [82], and it has been hypothesized that a cytokine storm and complement activation may play key roles in the development of AKI in critically ill children with COVID-19 [82]. On the other hand, AKI can also be associated with multisystem inflammatory syndrome associated with COVID-19 (MIS-C); the prevalence of AKI during MIS-C is between 15% and 28% [83,84]. The inflammatory response, led by IL-6 and IL-8, plays a crucial role in the development of AKI during MIS-C [83,84]. Other etiologies that may contribute to AKI in MIS-C are imbalanced renin-angiotensin-aldosterone system activation, endothelial dysfunction, complement and coagulation, obstruction of the glomerular capillaries by red blood cells, and drug toxicity [85].

Gastrointestinal diseases: acute appendicitis (AA) is an example of a gastrointestinal disease that may complicate AKI; during AA, AKI can in fact occur in 7.4% of patients [36]. Among patients with AA, AKI has been associated with the presence of vomiting, dehydration ≥ 5%, an axillary body temperature ≥ 38.5 °C, and higher levels of reactive c-protein and neutrophils [36]. In this case, both a pre-renal component with hypovolemia and the inflammatory response seem to play a role in AKI occurrence. AKI can also be present in patients with acute gastroenteritis (AGE); about one quarter (24.6%) of patients hospitalized for AGE may indeed suffer from AKI [37]. The duration of symptoms before hospitalization as an anamnestic, dehydration > 5% as clinical, and serum bicarbonate levels as biochemical parameters were significant and independent predictors for AKI in children hospitalized for AGE [37]. In this case, hypovolemia and dehydration are the main AKI causes. Finally, Bogari et al. also investigated the prevalence of AKI in a cohort of both adult and pediatric patients with AGE, reporting a prevalence of 1.6% in patients under 18 years of age [86], while Bradshaw et al. reported a prevalence of AKI of 0.8% in children hospitalized with diarrheal illness [87], and Mazankova et al. found that acute intestinal infections of viral etiology complicate AKI in 30–35% of cases [88].

Infectious disease: as pointed out in the former sections, AKI can be a complication of many infections in children, such as AA [36], AGE [86], CAP [34], acute bronchiolitis [35], and COVID-19 infection [82]. Malaria is another infectious disease that can lead to complications such as AKI, with an estimated 24–59% of African children experiencing AKI when afflicted with severe malaria [89]. Moreover, in a recent study examining pediatric patients with febrile urinary tract infections (fUTI), it was found that out of 849 children hospitalized for fUTI, 124 (14.6%) had AKI [38]. Notably, the prevalence of AKI surged to 30% when underlying congenital anomalies of the kidney and urinary tract were present, underscoring the potential for fUTI to complicate into AKI [38].

Hematological and oncological diseases: AKI can be present in pediatric patients with malignant diseases. For example, children with acute cancer can present kidney involvement with AKI manifestations. AKI can indeed occur in 16.9% of children with cancer, including 5.6% CA-AKI and 11.3% HA-AKI events [90]. Urinary system cancer, hepatic cancer, and retroperitoneal malignancies are the three types of cancer with the highest incidence of AKI in Chinese pediatric cancer patients [90]. Park et al. demonstrated that, among pediatric tumors, acute myeloid leukemia exhibited a higher 1-year cumulative incidence (88.4%) compared to other malignancies [91]. It was also shown that the three top risk factors for HA-AKI settings are respiratory failure, shock, and ileus, whereas the top three risk factors for CA-AKI are heart failure, epilepsy/convulsion, and shock [90]. Moreover, treatment with nephrotoxic agents contributes to the development of HA-AKI [90]. On the other hand, an example of a hematological disease that may complicate AKI is sickle cell disease (SCD). Little is known regarding the epidemiology of AKI in children with SCD. A recent study revealed an AKI incidence of 8% in patients admitted for acute chest syndrome [92]. Baddam et al. showed that AKI occurred in 17% of patients admitted for pain crises [93]. The etiology of AKI in children in these two studies is likely related to different causes, such as higher exposure to nonsteroidal anti-inflammatory drugs or repeated hypoxic ischemic episodes to the kidneys associated with vaso-occlusive crises [94].

Prematurity: AKI may also manifest in premature infants, as evidenced by the Preterm Erythropoietin Neuroprotection Trial’s examination of AKI incidence and impact among 932 extremely low-gestational-age neonates [95]. In this research, the occurrence of AKI stood at 38%, showing notable variations depending on gestational age, as AKI rates were notably elevated with decreasing gestational age and birth weight [95].

Cardiac diseases: The concept of cardiorenal syndrome, highlighting the reciprocal relationship between cardiac and renal dysfunction occurring simultaneously or sequentially in acute and chronic conditions, has been recently introduced in adults but has been rarely documented in pediatric populations [96]. In a retrospective epidemiological analysis involving pediatric intensive care unit patients, among 254 instances of AKI, 17% were observed in individuals with concurrent cardiac conditions, with roughly one-quarter of these cases being newborns diagnosed with congenital heart disease, exhibiting concomitant renal ischemia/reperfusion injury [41]. Furthermore, AKI frequently occurs as a complication following pediatric cardiac surgery, correlating with elevated levels of morbidity and mortality [97]. For example, Blinder et al. described an incidence of cardiac-surgery-associated AKI of 36% in children aged 0–36 months undergoing congenital cardiac surgery [30].

Nephrological causes of AKI: AKI can also be caused by many nephrological conditions, such as acute glomerulonephritis, nephrotic syndrome, vascular insults, or obstructive uropathies. Acute glomerulonephritis: any severe form of glomerulonephritis can present with AKI and rapidly progressive glomerulonephritis [18]. It has been reported that acute glomerulonephritis can be associated with AKI in 12% of cases [39]. Clinical signs usually include high blood pressure, swelling, often visible blood in the urine, and rapidly increasing levels of blood urea nitrogen and creatinine. Rapidly progressive glomerulonephritis resulting from postinfectious glomerulonephritis typically does not progress to CKD, whereas other types of glomerulonephritis, such as ANCA-positive glomerulonephritis, Goodpasture’s syndrome, and idiopathic RPGN, commonly present with AKI and may quickly develop into CKD, regardless of treatment [18]. Acute interstitial nephritis (AIN): AIN can lead to AKI due to a drug reaction or idiopathic causes [98]. Children with AIN may exhibit symptoms such as rash, fever, joint pain, eosinophilia, and pyuria, with or without eosinophiluria. Common medications associated with AIN include methicillin and other penicillin analogs, cimetidine, sulfonamides, rifampin, nonsteroidal anti-inflammatory drugs, and proton pump inhibitors, although other drugs can also cause AIN less frequently [98]. Nephrotic syndrome: children with nephrotic syndrome develop a variety of acute complications that can be serious and life-threatening, including infections, venous thromboembolism, and AKI [40]. AKI is attributed to prerenal azotemia from intravascular volume depletion in children with nephrotic syndrome. Risk factors for AKI include steroid-resistant nephrotic syndrome, infection, and nephrotoxic medication exposure [40]. Rheault et al.’s group described how hospitalized patients with nephrotic syndrome frequently experience complications from AKI, with an incidence of 50.9% [40]. Vascular insults: renal cortical necrosis, as a cause of AKI, is more prevalent in young children, especially newborns [18]. It is linked to hypoxic/ischemic events due to perinatal anoxia, placental abruption, and twin-to-twin or twin-to-mother transfusions, leading to the activation of the coagulation cascade [18]. Children and infants with cortical necrosis often exhibit gross or microscopic blood in the urine, reduced urine output, and may also experience hypertension [18]. Hemolytic uremic syndrome (HUS): HUS is one of the leading causes of AKI in children and a primary reason for chronic renal replacement therapy [99]. Keenswijk et al. reported a prevalence of AKI associated with HUS of 20.3% [39]. The most common infectious agents causing HUS are enterohemorrhagic Escherichia coli, which produce Shiga toxin and belong to the serotype O157, along with several non-O157 serotypes [99]. Classic clinical features of HUS include a triad of microangiopathic hemolytic anemia, thrombocytopenia, and AKI [99]. Obstructive uropathies: urinary tract obstruction can lead to AKI, with a reported prevalence of 6% [18,39]. Obstructions may be due to congenital abnormalities such as posterior urethral valves, bilateral ureteropelvic junction obstruction, or bilateral obstructive ureteroceles [18]. Acquired obstructions can occur from passing kidney stones or, in rare cases, tumors.

**Prevention of Pediatric Acute Kidney Injury:** The prevention of acute kidney injury (AKI) in the pediatric population is crucial due to the significant morbidity and mortality associated with this condition. Several pharmacologic strategies have been explored to mitigate the risk of AKI in various clinical settings.

**Furosemide and bumetanide:** furosemide and bumetanide are loop diuretics commonly used to manage fluid overload in critically ill pediatric patients [100]. While these agents can help maintain urine output and manage volume status, their role in preventing AKI is controversial [100]. Diuretics may delay the progression to severe AKI by reducing renal congestion and edema [101]. However, the overuse of loop diuretics can lead to volume depletion and electrolyte imbalances, potentially exacerbating renal injury [101]. Therefore, the use of furosemide and bumetanide should be carefully monitored and tailored to the individual patient’s needs.

**Dopamine:** low-dose or “renal” dose dopamine has been used historically with the intention of increasing renal blood flow and promoting diuresis [102]. However, in complex clinical situations, it may actually worsen renal perfusion [102]. Meta-analyses have shown that “renal-dose” dopamine does not benefit the prevention or improvement of AKI in critically ill children [103,104,105]. Consequently, dopamine is not recommended for AKI prevention in pediatric patients.

**Fenoldopam**: fenoldopam, a selective dopamine-1 receptor agonist, has been investigated for its potential renal-protective effects [106]. It is thought to enhance renal blood flow and reduce vascular resistance [106]. Some adult studies have suggested a benefit in reducing the incidence of AKI, particularly in high-risk surgical patients [106]. However, pediatric data are limited, and the efficacy and safety of fenoldopam for AKI prevention in children remain uncertain. Further research is needed to establish its role in this population.

**Rasburicase:** rasburicase is an enzyme used to manage hyperuricemia in pediatric patients at risk for tumor lysis syndrome [107]. By catalyzing the conversion of uric acid to the more soluble allantoin, rasburicase effectively reduces serum uric acid levels and the risk of AKI in children undergoing cancer treatment [107].

**Theophylline:** theophylline has been studied for its potential renal-protective effects, particularly in neonatal populations [108]. It is thought to act through adenosine receptor antagonism, which may help mitigate ischemia-reperfusion injury in the kidneys [109]. A recent meta-analysis of seven randomized controlled trials revealed that the administration of theophylline was linked to a significantly lower incidence of AKI [109]. However, its use is not yet widely adopted, and further large-scale studies are required to validate its efficacy and safety in broader pediatric populations.

Preventing AKI in pediatric patients involves a multifaceted approach, including the careful management of fluid balance, the avoidance of nephrotoxic agents, and the consideration of pharmacologic interventions where appropriate. While agents such as rasburicase have a well-established role in specific contexts, the use of others, such as furosemide, bumetanide, fenoldopam, and theophylline, requires cautious application and further research to fully elucidate their benefits. Optimizing these preventive strategies is essential to improving outcomes for children at risk of AKI.

## 9. Treatment

Before consulting a pediatric nephrologist, a general pediatrician can implement several measures to stabilize a pediatric patient with AKI and initiate preliminary treatment. AKI is a condition that requires prompt therapy due to its rapid progression and significant morbidity and mortality. Although there is no specific pharmacologic treatment for AKI, management focuses on reducing or eliminating kidney injury while providing support and strict control of hemodynamic, electrolyte, and acid-base abnormalities [110]. In severe cases, children with AKI may need renal replacement therapy to remove endogenous and exogenous toxins and to maintain fluid, electrolyte, and acid-base balance until renal function improves [110].

The initial step involves a rapid clinical assessment to determine vital signs, hydration status, and the patient’s symptoms. It is essential to establish intravenous access promptly for the administration of fluids and medications. In cases of hypovolemia, administering a bolus of normal saline (20 mL/kg) is advisable, followed by a reassessment of the patient’s condition [110]. Careful monitoring of fluid intake and output is critical to avoid fluid overload or depletion. On the other hand, in euvolemic children, total daily fluid intake should include insensible loss (300–500 mL/m^2^ per day), urine output, and potential other ongoing losses. Interestingly, volume status assessment is important as fluid administration is contraindicated in patients with fluid overload or heart failure. In children with fluid overload, fluid intake should be restricted, and the need for diuretics (such as furosemide) should be considered [110].

The Clinical Dehydration Scale (CDS) is a standardized tool utilized in pediatric clinical settings to assess the volume status of patients, particularly regarding dehydration [111]. This scale allows physicians to evaluate the degree of dehydration in pediatric patients systematically. It typically encompasses a set of clinical indicators, such as heart rate, capillary refill time, mucous membrane moisture, and skin turgor, among others [111]. By assessing these parameters, the CDS enables clinicians to categorize the severity of dehydration, ranging from mild to severe, aiding in the prompt and accurate management of pediatric patients presenting with dehydration [111].

Addressing electrolyte imbalances and acid-base disturbances is a priority. For hyperkalemia, intravenous calcium gluconate can be administered to stabilize cardiac membranes [110]. Additionally, insulin accompanied by glucose can facilitate intracellular potassium shift, and sodium bicarbonate may be used in the presence of concurrent metabolic acidosis. If significant and symptomatic acidosis is present, the administration of sodium bicarbonate is warranted [110].

Blood pressure management is crucial. Blood pressure should be monitored closely; treating hypertension with appropriate antihypertensive medications and managing hypotension with fluids and vasopressors, if indicated. Immediate discontinuation of nephrotoxic drugs is necessary, followed by a reassessment of the current pharmacological regimen [110]. In cases where infection is suspected, empirical antibiotic therapy should be initiated based on preliminary cultures and clinical data. Continuous monitoring, including frequent measurement of urine output—using a urinary catheter if necessary—is essential to track renal function. Serial blood tests should be performed to evaluate electrolytes, renal function (creatinine, BUN), and acid-base status. Nutritional support should commence with an appropriate diet that restricts protein and potassium intake while ensuring adequate caloric provision [110].

These interventions are fundamental to stabilizing the patient and preventing further renal deterioration while awaiting specialist consultation and management from a pediatric nephrologist.

The criteria for consulting a pediatric nephrologist, which align with the criteria for initiating dialysis, include several critical indicators that must be carefully assessed. These criteria are designed to guide healthcare professionals in determining the appropriate timing for a nephrology consultation and potential dialysis initiation.

Clinical judgment becomes paramount in making these decisions [112].

Specifically, the indications for consulting a pediatric nephrologist in patients with oligo-anuria, unresponsive to furosemide, include:

Hyperkalemia exceeding 6.5 mmol/L accompanied by T-wave changes on the ECG;
Severe fluid overload with evident pulmonary edema;
Urea levels surpassing 40 mmol/L (or exceeding 30 mmol/L in a newborn);
Pronounced hypo- or hypernatremia or acidosis;
Presence of multisystem failure.

Anticipation of prolonged oliguria, as seen in conditions such as hemolytic uremic syndrome, allows for the necessary space for potential blood transfusions and dietary adjustments [112].

## 10. Conclusions

AKI could have a significant impact on the lives of patients. Despite this, AKI is often underrecognized, especially in children. A correct AKI diagnosis, however, is pivotal, in the short term, to counteract the pathophysiological mechanisms, reduce morbidity and mortality, and, in the long term, to establish an adequate nephrological follow-up because patients who have presented with an AKI episode present an increased risk of CKD. Keeping in mind the key elements needed to diagnose AKI could help physicians reduce the instances of underdiagnosed AKI. Currently, a limitation in diagnostics is that creatinine is a late marker, and although new biomarkers exist, they have not yet been implemented in clinical practice. However, FENa may be useful in diagnosing intrinsic renal damage.

## Figures and Tables

**Table 1:** Advantages and disadvantages of the markers of AKI.

Marker	Advantages	Disadvantages
Serum Creatinine (SCr) and Urine Output	Widely available and inexpensive. Established benchmarks and guidelines (e.g., KDIGO) for diagnosis [2].	Delayed response to kidney injury. Influenced by extrarenal factors, leading to possible inaccuracies. Poor sensitivity and specificity in early AKI detection [2].
Cystatin C (Cys-C)	Not significantly influenced by muscle mass, age, or sex. Can detect changes in kidney function earlier than SCr [59].	Costlier and less readily available than SCr. Can be affected by thyroid disorders, obesity, and corticosteroid use [59].
Neutrophil Gelatinase-Associated Lipocalin (NGAL)	Increases rapidly after kidney injury (within 6 h). High sensitivity and specificity for AKI, especially in pediatric populations [64].	Elevated in conditions other than AKI (e.g., infections, inflammation). Higher baseline levels in cancer patients, potentially leading to false positives [64].
Kidney Injury Molecule-1 (KIM-1)	Highly specific for proximal tubular injury. Effective in distinguishing between different types of kidney injury [61].	Limited availability and higher cost. Requires further validation in diverse clinical settings [61].
Interleukin-18 (IL-18)	High sensitivity and specificity for acute tubular necrosis. Early marker of inflammation and injury [61].	Levels can be affected by various inflammatory and autoimmune diseases. Requires more extensive clinical validation [61].
Tissue Inhibitor of Metalloproteinases-2 (TIMP-2) and Insulin-like Growth Factor Binding Protein 7 (IGFBP7)	Early markers of cellular stress and G1 cell cycle arrest. Detectable in urine shortly after AKI onset [67].	Elevated levels may also be observed in diabetic patients. Costly and not widely available in all clinical settings [67].
Urinary Calprotectin	Increases significantly in AKI, especially in intrinsic AKI. Useful in distinguishing between pre-renal and intrinsic renal injury [66].	Limited use due to the need for further clinical validation. May be influenced by other inflammatory conditions [66].
Urinary Retinol Binding Protein (URBP4)	High sensitivity for renal tubular damage. Rapid normalization during recovery compared to SCr [72].	Requires additional research for widespread clinical application. Higher cost compared to traditional methods [72].
Liver-type Fatty Acid Binding Protein (L-FABP)	Promising biomarker for early detection and evaluation of AKI. Reflects renal hypoxia and oxidative stress [74].	Limited availability and higher cost. Requires further validation in larger clinical trials [74].
Clusterin	Early marker of proximal tubular injury. Anti-apoptotic effects, correlates with cell aggregation and adhesion [76].	Limited clinical use and availability. Further research needed for validation and standardization [76].
